# Social support experiences of adolescents living with perinatal HIV in rural Limpopo, South Africa

**DOI:** 10.4102/sajhivmed.v25i1.1521

**Published:** 2024-07-04

**Authors:** Rirhandzu A. Mabasa, Linda Skaal, Tebogo M. Mothiba

**Affiliations:** 1 Department of Public Health, Faculty of Health Sciences, University of Limpopo, Polokwane, South Africa; 2 Faculty of Health Sciences, University of Limpopo, Polokwane, South Africa

**Keywords:** adolescents, perinatal, HIV, social, support, experiences, rural

## Abstract

**Background:**

Adolescents with perinatal HIV (APHIV) experience emotional turmoil, which is worsened by real or perceived negative impacts on the adolescents’ relationships, aspirations for their careers, and aspirations for their families.

**Objectives:**

To explore the experiences of APHIV with regard to social support on their mental health and general well-being in the Vhembe District of Limpopo province.

**Method:**

A mixed-methods sequential exploratory design was employed to conduct in-depth one-on-one interviews in the Vhembe District of the Limpopo province of South Africa. The interviews were conducted in selected community health centres and clinics over a period of four months (April 2019 - July 2019). This study included APHIV between the ages of 10 years and 19 years who had been initiated on antiretroviral therapy before the age of 10 years.

**Results:**

Two major themes emerged. Theme 1 – Experiences within the family – included the sub-themes experience of positive social support within the family, and lack of support in the family. Theme 2 – Experiences outside the family – included the sub-themes experiences at the clinic, experiences at community level, and experiences at school and with friends.

**Conclusion:**

Adolescents with perinatal HIV are in need of social support from their loved ones as well as the community. Expansion of household programmes and intervention through integration of services by the multidisciplinary team might assist with alleviating the social support needs which will improve their mental health and adherence to treatment.

**What this study adds:** This study employed qualitative methods to establish a gap in the social support needs for APHIV in rural areas of Limpopo.

## Introduction

An increased focus on adolescents in response to the rise in the number of adolescents living with HIV (ALHIV) was found to accelerate better services and results.^1^ However, the rate of the development is still modest when compared to the expanding demands of ALHIV.^2^ The population of ALHIV continues to steadily rise globally, with an increase in the number of children who were perinatally infected with HIV reaching adolescence.^3,4,5^

Currently, sub-Saharan Africa, especially Eastern and Southern Africa (including South Africa), is estimated to have 1.74 million ALHIV, which is 60% of the global total.^6^ Adolescents living with perinatal HIV (APHIV) in sub-Saharan Africa experience numerous HIV-related challenges^7,8,9^ that limit their capacity to receive care, consequently impacting their mental and physical health.^10,11^ Previous studies have shown that APHIV experience multiple chronic illnesses, frequent hospital admissions and delayed milestones.^10,12^ Social issues within families, such as mental or physical illness, unstable housing situations, and drug or alcohol abuse, have a negative effect on the mental health of APHIV and also result in poor social support.^11^

Social support is defined as support from family, friends and others that is vital in improving the mental and physical health of the APHIV.^6^ Furthermore, as children, APHIV received support from caregivers or paediatric clinicians. This support fades as they grow to adolescence, leaving a gap in support needs.^13^ Zanoni et al. have stated that as adolescents transition from paediatric to adult care, the level of responsibility increases and they are often in conflict between requiring caregiver assistance and developing their independence, which results in difficulty supporting them socially and emotionally.^14^

In a quest to supplement the social support needs of APHIV and all ALHIV interventions such as support groups, teen clubs, and youth-friendly services, are aimed at improving the social and mental health of adolescents, especially APHIV.^15^ However, these interventions are based at health institutions and have proven to be ineffective in addressing social and emotional issues evidenced by the persistence of a high prevalence of mental health challenges among APHIV, which exceeds the HIV-negative adolescents by a factor of two to three.^16^

The majority of studies of ALHIV have focused on the challenges and adherence to antiretroviral therapy (ART),^17,18,19,20^ with very few studies highlighting the social and emotional support challenges and the impact thereof on the lives of APHIV, indicating the need for additional studies in this field.^14^ The aim of this study was to examine the experiences of APHIV in terms of social and emotional support and to assess the impact on their mental health and general well-being.

## Methods

A mixed-methods sequential explorative descriptive research design was employed to explore the experiences of adolescents who perinatally acquired HIV. It was conducted in 2019 for a period of four months. This project, adopted the qualitative, exploratory descriptive approach to explore and obtain in-depth social support experiences of APHIV in rural areas.

### Study setting

The study was conducted in 16 selected community health centres (CHCs) and clinics in Vhembe District, Limpopo province, South Africa. They comprised 13 clinics and 3 CHCs. These facilities were selected because they were accredited to roll out ART in 2015 and were managing APHIV. Vhembe District is primarily rural, with an unemployment rate of 70%, including youth. The district is divided into four local municipalities: Thulamela, Musina, Makhado, and Collins Chabane. There are two commonly spoken languages in the district: Xitsonga and Tshivenda. This study was conducted in three local municipalities: Thulamela, Musina, and Collins Chabane.

### Study population and sampling strategy

The population comprised APHIV who were between the ages of 12 years and 19 years, had started ART before the age of 10 years, and had collected ART on their own for six months from three sub-districts of the Vhembe District: Collins Chabane, Musina, and Thulamela. They were selected using the purposive sampling method. Sample size depended on data saturation, which was reached at 21 one-on-one interviews.

### Data collection

APHIV were identified and recruited telephonically through their parents or legal guardians. A face-to-face meeting was arranged to explain the objectives, the procedure during one-on-one interviews including voice recording as well as obtaining assent from APHIV below 18 years. Data were collected using one-on-one interviews in the two commonly used languages, using a semi-structured interview guide. Adolescents were asked open-ended questions and probed accordingly to obtain in-depth and broader experiences of social support from APHIV. Interviews were conducted in a less frequently used room to provide confidentiality and secrecy and they were voice-recorded, and field notes were noted.

### Data analysis

Data were analysed manually using Tesch steps of data analysis.^21^ Voice recordings were transcribed and translated to English for analysis. Manual analysis of the transcripts occurred through reading and jotting down ideas on the margins of each transcript. A list of all topics was written, and similar topics were grouped and organised into columns labelled as either major topics, unique topics or miscellaneous. Topics were organised into codes, and the best wording for each topic was identified. Topics were summarised into themes and sub-themes and sent to an independent coder for agreement on the final themes and sub-themes. The principal researcher and the independent coder agreed on the final themes and sub-themes.

### Trustworthiness

Credibility, dependability, transferability and confirmability were ensured throughout the research process. Credibility and confirmability were ensured by triangulation, member checks and examination of previous research findings. Dependability was ensured by describing research methodologies in detail, allowing a future researcher to replicate the work. Transferability was ensured by employing a full description of the qualitative research methodology, which included the research design, population, sampling method, data collection method, and data analysis.

### Ethical considerations

This research project was approved by the Turfloop Research Ethics Committee (TREC/228/2019: PG) and permission to conduct the study was obtained from the Limpopo Provincial and District Department of Health (reference numbers LP-201910-018 and S5/6). Signed assent and consent forms were sought from adolescents and their parent or guardians. Anonymity was ensured using codes instead of names, and confidentiality and privacy were maintained throughout the data collection process. Participants were told of their rights prior to the commencement of data collection.

## Results

### Description of the participants

Twenty-one participants were interviewed. Their ages ranged from 12 years to 19 years. The study was female dominated, with 17 female participants and 16 were in secondary school. There were eight double-orphaned participants who were cared for by grandmothers, siblings such as older sisters, aunts or other relatives.

Following thematic analysis, the following two themes emerged: experiences within the family, and experiences outside the family, which are presented in Table 1. Direct quotes from participants are presented in italics.

**TABLE 1:** Summary of the results.

Theme	Sub-themes
Experiences within the family	Positive social support in the family
	Lack of social support in the family
Experiences outside the family	Experience at the clinic
	Experience at the community
	Experience at school and with friends

### Experiences of adolescents living with perinatal HIV within the family in the context of social support

This theme focused on the experiences of APHIV within the family as a source of social support. The following sub-themes emerged: experiences of positive social support within the family and lack of social support within the family. Table 1 summarises the results.

### Social support within the family

According to the study’s findings, more adolescents cited that the social support of parents and loved ones helped them to be adherent to ART. The majority of those who had lost one or both of their parents, however, had negative emotional support from either family members or the parent who is still alive, particularly their fathers:

‘My sister supports me emotionally and financially; she also reminds me my treatment.’ (Particpant 007, 17 years old, female)

‘My mother works far, and I am staying with my granny. She is very supportive; she prepares food for me before taking my medication.’ (Participant 005, 14 years old, female)

‘My mother is my support system, she reminds me of the treatment dates and time.’ (Participant 008, 15 years old, female)

Adolescents staying with their parental relatives only received financial support but lacked emotional support:

‘My father buys food in the house, but he only buys food that he likes and sometimes he makes me angry that is why I refuse to wash the dishes.’ (Participant 001, 16 years old, female)

‘My father supports me financially and treats me well but he is in Gauteng and the people I stay with do not care about me or my condition, no one wants to know about my well-being or my medication.’ (Participant 013, 16 years old, male)

### Lack of support within the family

Adolescents who lived with people other than their biological parents or immediate family members like siblings encountered more familial difficulties. They lacked social and emotional support and endured emotional abuse:

‘I was constantly sick, sometimes I would not go to school because I was not well, my aunt would say I was not sick, I was lazy to do household chores. Sometimes she would tell her friends that she is not the one who killed my mother, AIDS killed her.’ (Participant 009, 17 years old, female)

‘My step-mother is ill-treating me. She has separated my eating utensils from the rest of the family because she said she does not want me to infect them [*her and her children*] with my HIV. She also tells other people my HIV status without my consent.’ (Participant 013, 16 years old, male)

### Experiences outside the family

This theme describes the experiences of APHIV in the social support context. Three sub-themes emerged, as described below.

#### Experiences at the clinic

Some adolescents were receiving emotional support from clinic nurses and the majority were treated well by the clinic staff. They received counselling and advice in relation to medication and behavioural practices:

‘Nurses are treating me well, there was a time when I wanted to stop my medication, and they advise me not to give up because it might mean the end of my life, it would be dangerous.’ (Participant 011, 17 years old, female)

‘Nurses treat me well, I have never experienced any problem, when I am sick, and they check everything and give me advice on how to take care of myself as an adolescent who is living with HIV infection.’ (Participant 006, 15 years old, male)

‘I feel at home when I am at the clinic, they treat me well … Well. I can say people are different and so are the nurses at the clinic, someday, you will find someone who is friendly and tries to make you feel at home, another day, you find someone who gives you medication only, but the overall support is good.’ (Participant 017, 15 years old, female)

#### Experiences of social support in the community

Most APHIV do not receive social support from the community and have never been stigmatised by people outside their families. However, they live with the fear of being stigmatised and discriminated against by community members due to living with HIV.

Participant 005 (14 years old, female) said: ‘HIV is “that illness” [*vuvabyi lebyiya*], it is not like any other disease’. When asked what ‘that’ illness means, she responded: ‘It is a disease which cannot be cured, and people look at you as if you are not human enough when you have it.’

Participant 001 (16 years old, female) added: ‘Those ones at my place they laugh at you when you are taking ART’.

#### Experiences at school and with friends

Adolescents had civil relationships with friends and teachers but did not get support from them on their HIV-positive status because they did not disclose it to anyone. They relate to one another normally:

‘I have a good relationship with everyone at school, but I have not told anyone about my HIV positive status, don’t expect to be supported by anyone because I am not sick.’ (Participant 002, 18 years old, male)

Due to the perceived stigma associated with HIV APHIV did not trust their peers when it came to disclosing their HIV-positive status. They believed that their friends would treat them badly or even laugh if they knew their HIV-positive status:

‘No, they would treat me differently, they would also gossip about me.’ (Participant 004, 13 years old, male)

‘I know my friends would laugh at you if they knew your HIV status forgetting that no one chooses to be sick.’ (Participant 005, 14 years old, female)

## Discussion

We documented the following experiences from APHIV in terms of social support: positive social support for APHIV living with their mothers or both parents, and lack of social support from guardians or fathers as single parents and relatives.

Our findings are similar to other studies, which revealed that APHIV lacked social and emotional support due to negative relationships with non-biological parents and this resulted in poor adherence to ART.^22^ Nsibandze et al. have stated that orphaned female APHIV cited that they rely on a higher power for emotional and social support, since they had no support from their families and communities.^23^ According to. Madiba and Mohlabane, lack of social and emotional support is associated with non-adherence to ART in the context of APHIV.^24^

Adolescents in our study were subjected to social and emotional abuse by their guardians. This abuse occurred in the form of verbal insults, disclosing the adolescent’s HIV-positive status to other people without their consent, and segregating APHIV utensils from the rest of the family.

Unlike orphaned APHIV who lived with guardians or relatives, APHIV who still had both parents were protected from emotional stress and received social support through the provision of basic needs and emotional support. These findings are similar to those found by Govender et al.^4^ and Madiba,^17,24^ who reported that APHIV reported receiving positive social support from their families through being supervised, reminded to take medication, and having their needs taken care of.

Some nurses at the healthcare facilities offered emotional and social support by providing health information related to HIV and conducting mental health screening. However, some APHIV reported only receiving medication during their clinic visits. This could be due to a lack of skill, as found by Woollett,^25^ who asserted that a lack of skilled mental health workers could leave a gap in the mental healthcare of patients.

Adolescents in our study had positive social relations with their peers and teachers. They were able to have friends and, according to them, relate well with everyone, which gave them a sense of belonging and acceptance. Tumwine, Aggleton and Bell^26^ reported that affiliative support in the form of new friends provides APHIV with a sense of belonging. However, APHIV in our study did not disclose their HIV status to their friends and teachers due to a fear of being stigmatised and isolated.

HIV remains a highly stigmatised disease^27,28,29^ and the fear of stigma and social isolation leads to non-disclosure and, consequently, non-adherence to treatment and lack of social support.^17^ According to Felker-Kantor et al.,^30^ HIV-related stigma has significant association with mental health disorders such as depression and anxiety. Armoon et al.^11^ reiterate that HIV secrecy and stigma become a part of the lives of APHIV, to the extent that they keep that part of their lives a secret.

Our findings are consistent with other studies where APHIV reported being afraid of disclosing their HIV status to friends and relatives due to the fear of stigma and discrimination expressed by the community towards people living with HIV.^3,12^ McHenry et al. reported that myths and misconceptions about HIV, such as HIV diagnosis being linked to sexually immorality, contribute to stigma and discrimination.^31^

### Limitations

Our study has limitations that should be considered. This study was conducted in one district of the Limpopo province, which is primarily rural. This means that the results may not be generalisable to urban settings. Some APHIV were unable to articulate themselves well due to their young age. Finally, we did not explore the challenges faced by caregivers, especially non-biological parents taking care of APHIV, who could have offered insight on their own experiences.

## Conclusion

We highlighted the social experiences of APHIV which might affect their management and outcomes such as poor adherence to ART, quality of health as well as their lifespan. We found that family social support remains vital for APHIV. We recommend family-orientated interventions to improve their social support experiences. Furthermore, there should be strengthening of a multidisciplinary approach to enhance and strengthen family relations.
